# Corneal surface reconstruction for the chemical injured eye by transplanting autologous cultivated limbal epithelial sheet “Nepic^®^”

**DOI:** 10.20407/fmj.2024-031

**Published:** 2025-04-17

**Authors:** Daisuke Kato, Koji Hirano, Atsuhiro Tanikawa, Yasuki Ito

**Affiliations:** 1 Department of Ophthalmology, Fujita Health University, School of Medicine, Toyoake, Aichi, Japan; 2 Department of Ophthalmology, TOYOTA Memorial Hospital, Toyota, Aichi, Japan; 3 Department of Ophthalmology, Fujita Health University Bantane Hospital, Nagoya, Aichi, Japan

**Keywords:** Chemical Injury, Cultivated Limbal Epithelial Cell Sheet Transplantation (CLET), Nepic^®^, Unilateral Limbal Stem Cell Deficiency

## Abstract

**Background:**

“Nepic^®^” (Japan Tissue Engineering Co., Ltd., Gamagori, Japan) is an autologous cultivated limbal epithelial sheet, approved in 2020 in Japan for the reconstruction of the corneal surface in cases of limbal stem cell deficiency (LSCD). Because the surgical procedure known as cultivated limbal epithelial cell sheet transplantation (CLET) has only recently been introduced and the number of cases remains limited, accumulating clinical experience is essential to ensure the safety and success of this procedure. Herein, we report the clinical course of a patient with unilateral LSCD who underwent CLET for a corneal surface reconstruction using Nepic^®^.

**Case presentation:**

A 50-year-old man sustained bilateral eye injuries from mortar during construction work. The patient’s right cornea exhibited LSCD. Initial treatment involved a 360° limbal transplantation with an allo-corneal graft at a university hospital. However, graft rejection occurred, and the corneal surface was subsequently covered by conjunctival tissue within 2 months. Because the patient’s left cornea and conjunctiva appeared healthy, we performed CLET with Nepic^®^ 3 years after the limbal transplantation.

**Conclusions:**

When using Nepic^®^, it is essential to become accustomed to handling the cultivated epithelial sheet on the ring-shaped holder; however, cultivated corneal epithelium transplantation can also be performed without the carrier material, but rather as a sheet. Though the corneal surface appeared smooth and shiny at 7 months postoperatively, we seek to determine whether the epithelial cells on the patient’s cornea are of corneal or conjunctival origin without resorting to invasive procedures, such as biopsy.

## Introduction

The limbal papillary structure, known as the palisade of Vogt, has been identified as a regenerative organ for corneal epithelial cells through immunohistochemical studies.^1,2^ Damage to these limbal stem cells, termed limbal stem cell deficiency (LSCD), impairs the regeneration of normal corneal epithelial cells and can lead to significant visual disturbances due to a hazy and irregular corneal surface.^3^

For patients with unilateral LSCD, such as those with chemical or thermal injuries to the cornea, auto-limbal transplantation from the uninjured eye can restore corneal function.^4–6^ However, this procedure necessitates a large limbal graft from the healthy eye, which may result in LSCD in the donor eye.^3,7^

Advancements in tissue engineering have introduced cultivated corneal limbal transplantation, a technique that reduces the amount of tissue taken from the uninjured eye, thereby lowering the risk of rejection. Successful applications of this method have been reported since approximately the year 2000.^8–10^ In the early 2000s, we attempted autologous cultivated limbal epithelial cell sheet transplantation (CLET) to reconstruct a unilateral thermal burn injury, following the procedure outlined by Pellegrini et al.^8^ Unfortunately, the procedure was unsuccessful because the stratified corneal epithelial sheet broke while on the culture dish during its removal with dispase, causing the cultured epithelial cells to fall apart due to the enzyme’s effect on cell-cell junctions.^11^

To address the challenges associated with handling multilayered corneal epithelial sheets after removal from the culture dish, several methods have been investigated. These include using fibrin gel,^12^ amniotic membrane as a carrier,^9^ and temperature-responsive cell culture surfaces.^13,14^

“Nepic^®^” (Japan Tissue Engineering Co., Ltd., Gamagori, Japan) is an autologous cultivated limbal epithelial sheet prepared following the methods outlined by Pellegrini et al.^8^ The stratified structure of the corneal epithelial sheet is maintained using a temperature-responsive cell culture surface, as reported by Nishida et al.^13,14^ Licensed in Japan in 2020 for the reconstruction of corneal surfaces affected by LSCD, Nepic^®^ has been utilized in clinical practice. However, owing to the limited number of cases where it has been applied, several questions remain regarding its application, such as the optimal stage of LSCD for using Nepic^®^, and the long-term prognosis of the transplanted epithelial sheet.

To enhance the safety and success of the procedure, accumulating clinical experience is crucial. Therefore, we report the clinical course of a patient with unilateral LSCD resulting from thermal injury, who underwent corneal surface reconstruction using Nepic^®^.

## Case report

### Patient’s history

A 50-year-old man sustained bilateral eye injuries from mortar during construction work. Initial first aid involved washing the eyes with tap water, followed by treatment with antibiotic eye drops administered by the nearest ophthalmologist. Despite these measures, the right corneal epithelial defect persisted. After 6 months, the right cornea was covered by conjunctival tissue, leading to conjunctival sac shortening, whereas the cornea and conjunctiva of the left eye appeared almost healthy. Diagnosed with unilateral LSCD due to chemical injury, the patient underwent a 360° limbal transplantation with an allo-corneal graft in the right eye at a university hospital 1 year after the injury.

At 1 month post-transplantation, the best corrected visual acuity (BCVA) of the right eye had improved from 0.01 preoperatively to 0.2. However, 3 months later, the visual acuity had reverted to 0.01 due to graft failure, despite treatment with immunosuppressive therapy, including ciclosporin (Neoral^®^) and betamethasone eye drops. The patient was subsequently referred to Fujita Health University Hospital for follow-up care of the right eye following graft rejection at the previous hospital.

**Figure 1:**
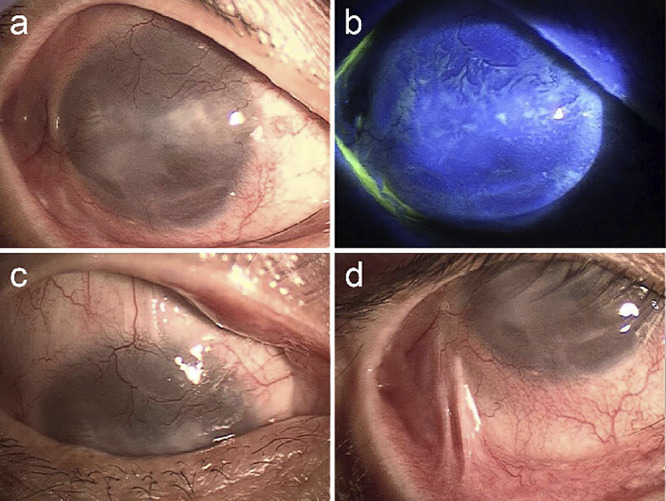
Corneal and conjunctival findings of the right eye before cultivated limbal epithelial cell sheet transplantation a. The conjunctival tissue covers almost the entire corneal surface, and the cornea is cloudy. b. No epithelial defect is observed in the fluorescent staining; however, the fluorescent shades indicate that tissue other than corneal epithelium covers the corneal surface except for the lower central area. c, d. The conjunctival strand seen in the upper and lower areas indicates conjunctival sac shorting.

Upon initial examination, the patient’s BCVA was 0.01 (uncorrectable) in the right eye and 0.3 (1.2×−1.5 D, cylinder −1.0 D, axis 10) in the left eye. Intraocular pressure could not be measured in the right eye, and it was 8 mmHg in the left eye. Although a smooth corneal surface was observed centrally in the right eye, conjunctiva with vessels had encroached upon the limbus from all directions, and corneal stromal opacification had begun. To manage this, the patient was instructed to use topical corticosteroids, either 0.1% betamethasone or 0.1% fluorometholone eye drops, to prevent further corneal opacity. Despite this treatment, the conjunctiva progressively invaded and covered almost the entire corneal surface over the following year, reducing the visual acuity of the right eye to hand movements (Figure 1).

In 2020, Nepic^®^ was licensed as a therapeutic material. We proceeded with reconstructing the ocular surface using Nepic^®^. Approval for this new therapeutic application was obtained from the Institutional Review Board at Fujita Health University Hospital, in accordance with the Declaration of Helsinki. Written informed consent for the publication of this case report and accompanying images was obtained from the patient.

### Surgery

Approximately 3 years after the initial limbal transplantation, CLET was performed on the right eye at Fujita Health University Bantane Hospital. At 4 weeks before the CLET procedure, a 2 mm×3 mm full-thickness limbal tissue sample was harvested from the 11 o’clock position of the limbus in the healthy left eye. This tissue was sent for cultivation at Japan Tissue Engineering Co., Ltd.

**Figure 2:**
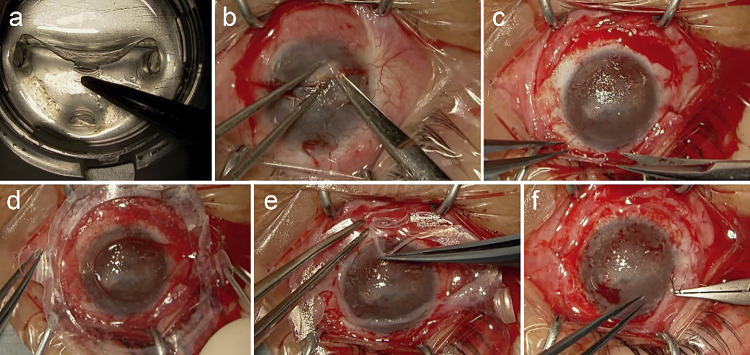
Intraoperative findings a. The cultivated corneal sheet was prepared for use prior to the initiation of surgery. Lowering the temperature allows the sheet to be readily peeled off the dish. b. The abnormal tissue that covered the corneal surface was removed bluntly using scissors. c. Exposed corneal surface. d. The cultivated corneal epithelial sheet (Nepic^®^) was placed on the corneal surface. e. Removal of the sheet from the holder is the most crucial part of this surgery. One should be careful not to create breaks in the sheet. f. The sheet was sutured using the 10-0 nylon running suture.

During the surgery, the cultivated corneal epithelial sheet was pre-removed from the culture dish (Figure 2a). Abnormal tissue covering the corneal surface of the right eye was peeled off with scissors (Figure 2b) but not resected, exposing the surface of the hazy cornea (Figure 2c). Subsequently, the sheet was brought with a ring-shaped frame and placed on the exposed corneal surface (Figure 2d), and the frame was removed by pulling out the four protrusions (Figure 2e). Once the epithelial sheet was positioned on the corneal surface, it was prone to tearing if any tangential movement occurred, making this the most challenging part of the surgery. The sheet was sutured to the limbus with a running suture of 10-0 nylon (Figure 2f), and the cut edge of the conjunctiva was stitched using 8-0 Vicryl^®^ to cover the sheet on the exposed sclera. The absence of defects in the epithelial sheet was confirmed using fluorescence, and a soft contact lens was placed on the corneal surface. Postoperatively, the patient was treated with topical 0.1% betamethasone four times daily for 6 months, which was then tapered to twice daily.

### Postoperative course

**Figure 3:**
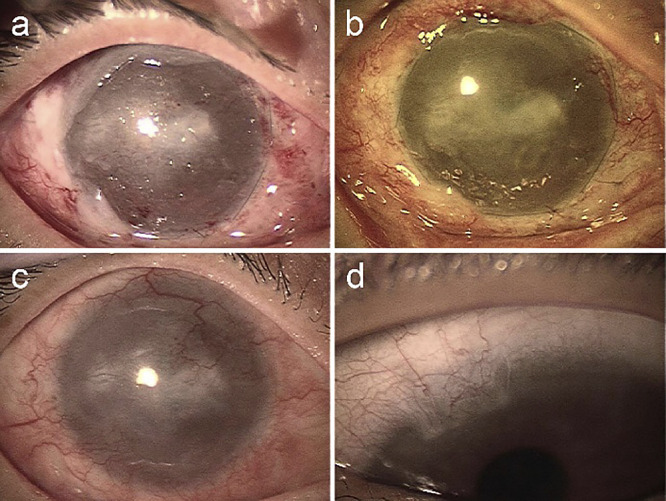
Postoperative appearance recorded by diffuse illuminated photos a. On the day following the surgery, an uneven corneal surface is seen after the removal of the contact lens. b. Ten days after surgery, the smooth corneal surface can be seen. c. At 7 months postoperatively, the shiny appearance of the corneal surface is preserved, but the invaded vessels are more prominent, and the corneal opacity in the central cornea is still seen. d. The 11 o’clock limbal area of the left eye is covered by the conjunctival tissue at 7 months postoperatively, or 8 months after the resection of the limbal tissue for cultivation.

**Figure 4:**
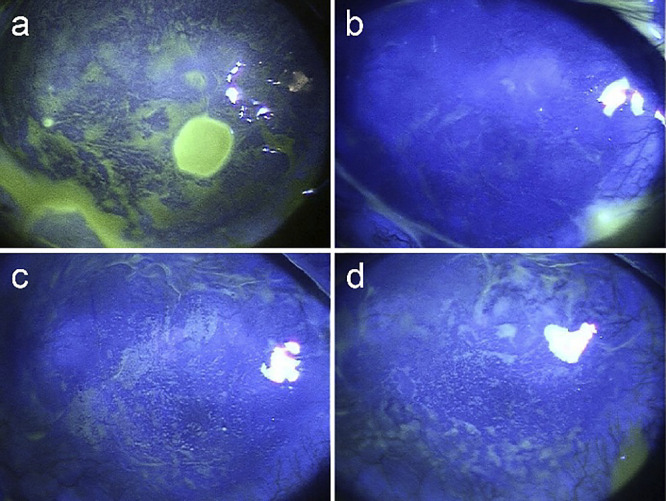
Corneal fluorescent stained appearances after surgery a. At 10 days postoperatively, a round-shaped epithelial defect is present in the nasal inferior area. b. The epithelial defect resolved 20 days after, and a shiny epithelial appearance can be observed. c. At 17 weeks postoperatively, diffuse superficial punctate keratopathy is seen in a random array. d. At 7 months postoperatively, a smooth corneal surface can be seen by slit lamp examination, but an uneven fluorescent pattern is observed in the upper and lower corneal surfaces.

The day following surgery, an uneven corneal surface appeared when the contact lens was removed during examination (Figure 3a). At 10 days postoperatively, a shiny corneal surface was observed (Figure 3b), although the central cornea remained cloudy, and an oval-shaped epithelial defect, 2 mm in diameter, was observed in the nasal region of the central cornea (Figure 4a). At 7 months postoperatively, the corneal surface remained shiny and smooth (Figure 3c), and the visual acuity in the right eye was 0.01. In the left eye, the 11 o’clock limbal area, from where the healthy limbal tissue was harvested, was covered by conjunctival tissue (Figure 3d), usually occluded by the upper lid, with a visual acuity of 0.1 (1.2×–2.25 D=cyl–1.0 D axis 20).

Figure 4 illustrates the postoperative course of the cornea by fluorescein staining. The epithelial defect observed 10 days after surgery (Figure 4a) resolved approximately 20 days later without any additional treatment, and no centripetal movement of epithelial cells was detected by the shade of the fluorescein during repair (Figure 4b). At 17 weeks postoperatively, superficial punctate epithelial defects appeared randomly on the corneal surface, not in a whirlpool array (Figure 4c). Despite the smooth appearance of the corneal surface under diffuse illumination observed in Figure 3c, the distribution pattern of the fluorescein in the upper or lower areas of the cornea differed from that in the central area at 7 months postoperatively (Figure 4d).

## Discussion

The temperature-responsive culture dish enabled the harvesting of cultured corneal epithelium as a stratified sheet without damaging cell-cell junctions or using fibrin glue, making the transplantation of the cultivated epithelium easier. This biologic carrier-free method may reduce the risk of infection.^13,15^ However, the cultured sheet is relatively fragile compared with those on carrier materials.^9,12^ During our surgery, we could place the cultured corneal epithelium on the exposed corneal surface as a sheet (Figure 2f). Nevertheless, care was needed to avoid tearing the sheet, particularly when removing it from the culture dish (Figure 2a) and placing it on the exposed corneal surface (Figure 2d). Although the ring-shaped holder with four protrusions (Figure 2a) facilitates the transfer of the cultured sheet from the dish to the patient’s cornea, careful handling is necessary to prevent tearing during removal from the protrusions. We must become accustomed to this procedure.

The corneal surface, which appeared uneven the day after surgery (Figure 3a), became smooth after 10 days (Figure 3b). Although a round-shaped epithelial defect was observed simultaneously (Figure 4a), it healed spontaneously after 3 weeks without showing a spiral pattern of fluorescein distribution (Figure 4b). It appears that the wound healing of the transplanted epithelium on the LSCD eye differs from that of a healthy eye.^14^ At 7 months postoperatively, a smooth corneal surface was observed under slit lamp examination (Figures 3c, 4d). However, it remains unclear whether the epithelium covering the corneal surface is of corneal or conjunctival origin, and we would like to determine this without invasive procedures such as a biopsy.

Corneal surface reconstruction using Nepic^®^ is considered a therapeutic keratoplasty and is not aimed at vision recovery. Even so, if autologous cultivated corneal epithelial transplantation could be applied earlier, better visual acuity post-surgery might be possible. The patient obtained a BCVA of 0.2 in the right eye 1 month after allo-limbal transplantation, which decreased to hand motion with the progression of limbal graft failure. The visual acuity postoperatively with Nepic^®^ remained 0.01 (non-correctable), possibly due to corneal scarring and lipid degeneration over the 3 years, leading to corneal opacity and irregular astigmatism. We are planning deep anterior lamellar keratoplasty in the patient’s right eye to obtain a clear cornea and correct the corneal astigmatism. This case raises the question of the ideal timing for corneal surface reconstruction using Nepic^®^.

## Conclusions

When using Nepic^®^, it is essential to become accustomed to handling the cultivated epithelial sheet on the ring-shaped holder. Despite this, this method does allow for the transplantation of the cultivated corneal epithelium as a sheet without the need for biologic material. The epithelial wound healing in the cultivated corneal epithelium occurs differently from that in healthy corneal epithelium. While the corneal surface after transplantation achieved a smooth and shiny appearance 7 months postoperatively, we would like to determine whether the epithelial cells on the patient’s cornea are of corneal or conjunctival origin without resorting to invasive procedures such as a biopsy.
